# Diffuse hematoma caused by spontaneous rupture of a parathyroid adenoma: a case report^☆^

**DOI:** 10.1016/j.bjorl.2017.06.006

**Published:** 2017-07-07

**Authors:** Lifeng An, Tiefeng Ji, Lin Li

**Affiliations:** a China-Japan Union Hospital of Jilin University, Department of Otorhinolaryngology Head and Neck Surgery, Changchun, China; b First Hospital of Jilin University, Department of Radiology, Changchun, China

## Introduction

A diffuse cervical hematoma attributable to spontaneous rupture of a parathyroid adenoma is uncommon. Although most such patients are hemodynamically stable, all of serious hemorrhage, dyspnea, and death are possible.^1^ In the present paper, we report a case of a diffuse cervical hematoma caused by spontaneous rupture of a parathyroid adenoma. Parathyroid adenomectomy was performed after the hematoma became absorbed after a period of normal breathing. We found that the bleeding expanded readily due to the absence of a protective anatomical barrier (muscles or ligaments) around the thin parathyroid membrane.

## Case report

A 61-year-old female presented to our otorhinolaryngological department 2 days after an ultrasonographic thyroid check-up, with progressive, prominent diffuse neck ecchymosis and mild dyspnea. She also exhibited odynphagia, hoarseness, and a slight fever. The butterfly-shaped ecchymosis extended from the upper neck to the nipple. There was no history of trauma, invasive manipulation, or infection.

**Figure 1:**
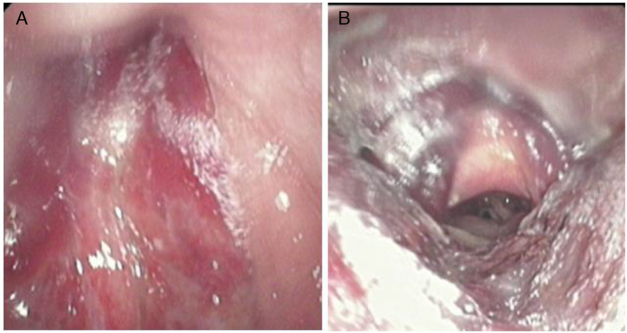
An extensive submucosal hemorrhage. (A) The nasopharynx; (B) the larynx and hypopharynx.

**Figure 2:**
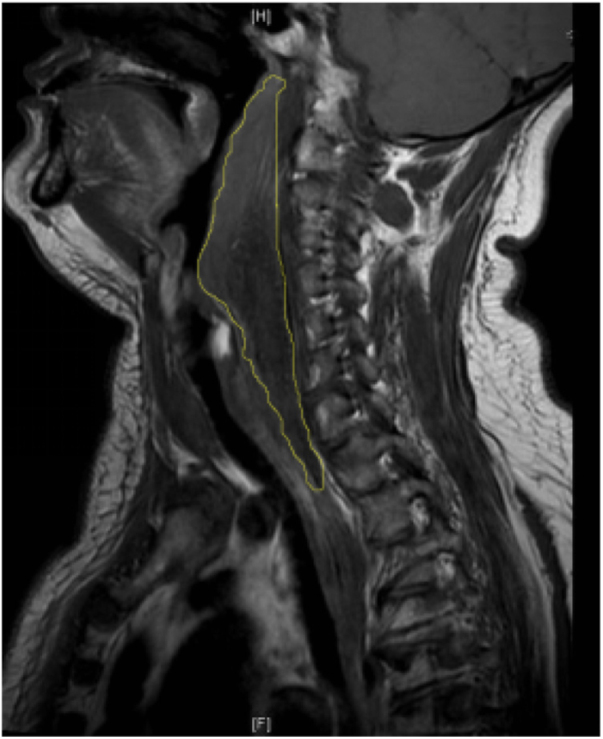
Sagittal magnetic resonance imagery revealed an abnormal irregular signal extending from the second cervical vertebra to the posterior mediastinum.

**Figure 3:**
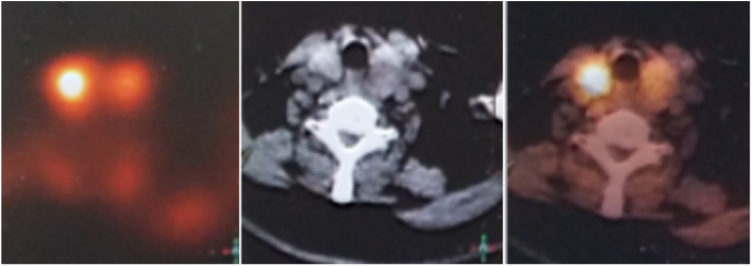
Single-photon emission computed tomography scans revealed a left-side parathyroid gland adenoma.

Ultrasonography revealed a 39.3 × 24.5 × 20 mm-sized hypoechoic area in the upper pole of the left-side thyroid gland where a clear-edged 36 × 18 × 12 mm hypoechoic area had been noted prior to the onset of illness. Laryngoscopy revealed an extensive submucosal hemorrhage extending from the nasopharynx to the hypolarynx and trachea (Fig. 1). Magnetic Resonance Imaging (MRI) yielded an abnormal irregular signal within the subcutaneous tissue and the inter- and intra-muscular spaces, extending from the posterior pharyngeal wall into the posterior mediastinum, with compression of the trachea and esophagus (Fig. 2). [99m]c pertechnetate scintigraphy did not evidence any parathyroid gland involvement, but a low-density lesion 33.2 × 16.5 mm in size was found in the dorsal region of the left thyroid (Fig. 3). No abnormal radioactive aggregate was apparent. We strongly suspected a spontaneous hematoma developing from a huge parathyroid tumor. Laboratory data were as follows: Parathyroid Hormone (PTH) 64.65 pg/mL (normal, 15–65 pg/mL), calcium 2.24 mmoL/L (normal, 2.10–2.60 mmoL/L), thyroid-stimulating hormone 0.9 mIU/L (normal, 0.37–4.94 mIU/L), free tri-iodothyronine 3.12 pmoL/L (normal, 3.10–6.80 pmoL/L), and free thyroxine 13.9 pmoL/L (normal, 12.0–22.0 pmoL/L).

**Figure 4:**
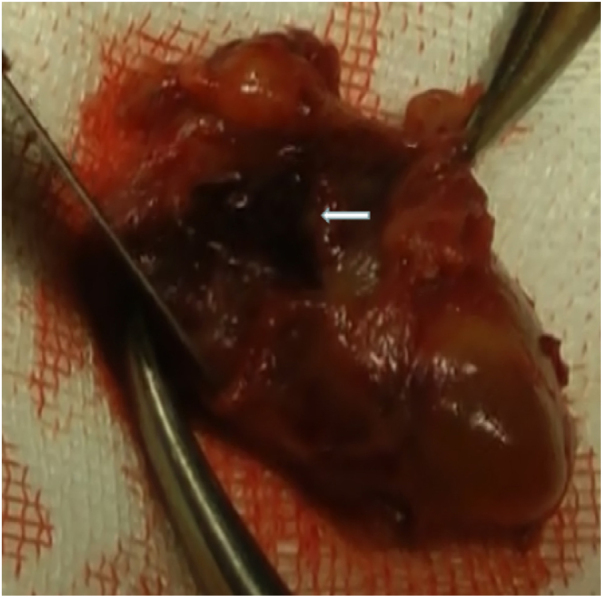
A clot within the tumor capsule.

Parathyroid adenomectomy was performed when the dyspnea had resolved and the ecchymosis had become brown in color. Interestingly, blood clots were found both next to the parathyroid tumor and within the tumor (when we cut the tumor capsule) (Fig. 4); however, we could not find the broken end of the vessel. Histological analysis identified a parathyroid adenoma.

The patient was discharged 5 days after the procedure. The levels of both PTH and calcium were measured again, and were within normal limits. At the 6-month follow-up, all symptoms had disappeared. Laryngoscopy yielded no sign of hemorrhage. The patient signed a consent form allowing us to present her data in this case report. Our local ethics committee also approved publication.

## Discussion

Cervical hemorrhage may be caused by subacute thyroiditis, dissection of an aortic aneurysm, a penetrating aortic ulcer, or rupture of a mediastinal mass or cyst. Parathyroid hemorrhage is rare but should not be ignored.^2^ Both neck and chest trauma, and vascular causes, should be considered. The first case of spontaneous parathyroid gland hemorrhage, reported in 1934, was unfortunately fatal.^3^ Hyperplasia, an adenoma, or other cancers of the parathyroid may cause intra- and extra-capsular hemorrhage.^4^ In the present report, we describe an extracapsular hemorrhage that extended widely throughout the subcutaneous, submucosal, and intermuscular spaces, caused by a huge parathyroid adenoma. Imaging techniques including ultrasonography, MRI, and/or Single-photon Emission Computed Tomography (SPECT), and an analysis of the patient's history are required for diagnosis when a cervical hematoma presents with various clinical manifestations including pain, dysphagia, dyspnea, a cervical mass, and ecchymosis.^5^ Differential diagnoses should be also considered. The serum calcium and/or PTH levels allow evaluation of parathyroid status. An aortic aneurysm, a penetrating aortic ulcer, or rupture of a mediastinal mass or cyst may be evident on ultrasound and/or other images.

In our current case, ultrasonography showed that the hypoechoic area in the left-side thyroid was significantly larger than at disease onset, and the edge was unclear (the edge had been clear prior to illness). We thus strongly suspected that a hematoma had developed from the tumor in the left-side thyroid. MRI was used to reveal the co-existence of a hematoma and an adenoma.

Parathyroid bleeding occurs most often in patients with adenomas.^6^ The extracapsular hemorrhage of our case may be attributable to the fact that both the huge tumor and the thyroid gland have relatively thin, weak capsules. The hematoma spread readily to the neck, the facial spaces, and the chest because the parathyroid gland is not sheathed by ligaments, muscles, or bones. If a hematoma spreads to the mediastinum, the symptoms may even mimic those of a dissecting aortic aneurysm or descending necrotizing mediastinitis, and the condition may be fatal.^7^

Conservative therapy was successful when used to treat the acute phase of the present condition; a similar result has been reported by van den Broek et al.^8^; the cited authors surgically excised the tumor after bleeding had stopped, to avoid recurrence of hemorrhage. No well-accepted guideline for the optimal timing of surgery exists. Chaffanjon suggested that surgery 3 months after the development of hemorrhage is optimal if the anatomical structure is clear.^9^ Delayed surgery may reduce the risk of surgical complications, such as incomplete tumor excision and injury to the recurrent laryngeal nerve. In our case, we performed surgery after the hematoma had become resorbed to some extent, at which time the anatomical structure was sufficiently clear to allow us to avoid complications.

A diffuse hematoma attributable to spontaneous rupture of a parathyroid adenoma is rare, but, in our experience, should be strongly suspected if a patient presents with a combination of symptoms (pain, dysphagia, dyspnea, a cervical mass, and ecchymosis); a parathyroid adenoma; and no history of trauma or surgery. Conservative therapy is appropriate in the acute phase in which the timing of a later operation is determined by the condition of the patient.

## Conclusion

We recommend that possible spontaneous rupture of a parathyroid adenoma should be suspected if a patient presents with a non-traumatic cervical hemorrhage.

## Conflicts of interest

The authors declare no conflicts of interest.
